# Psychological stress exposure to aged mice causes abnormal feeding patterns with changes in the bout number

**DOI:** 10.18632/aging.101320

**Published:** 2017-11-09

**Authors:** Chihiro Yamada, Sachiko Mogami, Tomohisa Hattori

**Affiliations:** ^1^ Tsumura Research Laboratories, Tsumura & Co., Ibaraki, Japan

**Keywords:** meal pattern, meal microstructure, aged mice, bout, stress

## Abstract

Stress responses are affected by aging. However, studies on stress-related changes in feeding patterns with aging subject are minimal. We investigated feeding patterns induced by two psychological stress models, revealing characteristics of stress-induced feeding patterns as “meal” and “bout” (defined as the minimum feeding behavior parameters) in aged mice. Feeding behaviors of C57BL/6J mice were monitored for 24 h by an automatic monitoring device. Novelty stress reduced the meal amount over the 24 h in both young and aged mice, but as a result of a time course study it was persistent in aged mice. In addition, the decreased bout number was more pronounced in aged mice than in young mice. The 24-h meal and bout parameters did not change in either the young or aged mice following water avoidance stress (WAS). However, the meal amount and bout number increased in aged mice for 0–6 h after WAS exposure but remained unchanged in young mice. Our findings suggest that changes in bout number may lead to abnormal stress-related feeding patterns and may be one tool for evaluating eating abnormality in aged mice.

## INTRODUCTION

Exposure to stress often causes eating abnormalities. Stress is known to either stimulate or suppress eating behaviors. Stress-related overeating and anorexia are recognized as symptoms in patients diagnosed with major depression [1], anorexia nervosa, and bulimia nervosa [2]. Even in research studies, applying psycho-logical stress to rodents can positively or negatively regulate food ingestion. For example, restraint stress and novelty stress induce decreased food intake [3–5], whereas social-defeat or repeated stress causes hyperphagia [6–8].

Currently, assessment of food consumption is often used as an indicator of appetite, but changes in feeding behavior may also be parameters that reflect appetite. Several studies investigating changes in feeding patterns using several feeding-related factor-administered mice and knock-out (KO) mice have been reported [9, 10].

Microstructural analysis of feeding behavior may be one of the most important tools. Feeding behavior consists of not only meal amounts, but also basic behavioral units that comprise a series of processes such as biting, chewing, and swallowing [11]. The “bout” parameter was defined in a previous paper [12] as a measurement of the smallest feeding behaviors such as nibbling and swallowing. It seems likely that such as assessment of bout parameters may reflect digestive tract function more delicately than meal amount. However, few studies on eating parameters focusing on the bout parameter have been conducted, and there have been no reports on the effects of stress.

The elderly are often exposed to social stressors such as a lack of social networks, divorce, and spouse bereavement. This degree of susceptibility to stress from social and environmental changes in the elderly has been demonstrated in research studies. In aged mice, stress due to environmental change causes persistent activation of the HPA axis when compared to young mice and we demonstrated that aged mice are clearly more vulnerable to stress than young mice and cause a sustained decrease in food intake [13, 14]. Additionally, since the elderly experience disease complications, such as homeostatic abnormalities and deterioration in oral functioning and swallowing ability [15], they may have different reactions depending on the type of exogenous stressor [16, 17]. Thus, eating abnormalities in elderly individuals exposed to stress may also lead to different phenotypic expressions compared to those of younger individuals. In addition, as a result of comparing the feeding patterns of young and aged mice, we found a characteristic feeding behavior that the number of bout increases in aged mice leading to the maintaining meal amount as a same level of young mice [18]. However, details on the characteristic feeding pattern in eating abnormalities and its mechanisms in the elderly exposed to stress have not been sufficiently studied.

Our ultimate goal is to confirm whether evaluating the characteristic feeding parameter “bout” in aged mice may be a tool to elucidate the mechanism of stress-induced eating abnormalities or the mechanism of development of therapeutic agents. As a first step, we examined the effect of stress-load (novelty environ-mental stress: novelty and water avoidance stress: WAS) in aged mice on the bout parameter by microstructure analysis of feeding patterns.

## RESULTS

### Voluntary movement

**Figure 1:**
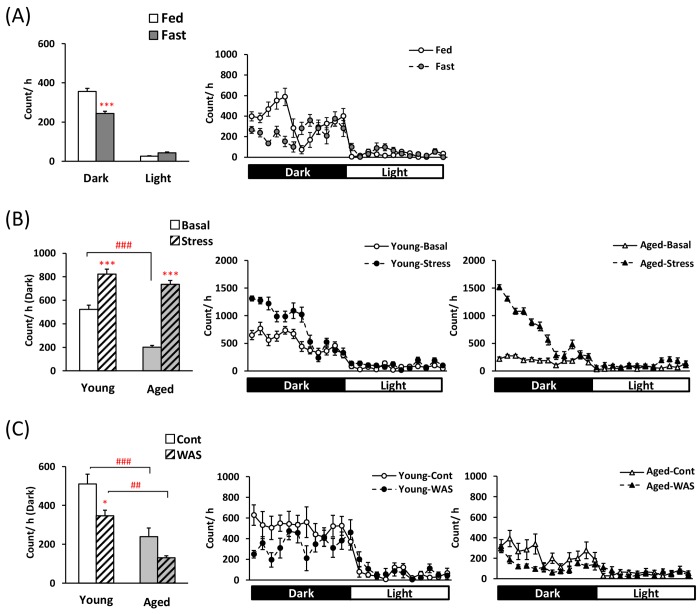
Changes in locomotor activity in young and aged mice after fasting or exposure to novelty stress/water avoidance stress (**A**) left: average voluntary movements after 24-h fasting in the dark/light phase, right: the 24-h changes after 24-h fasting in young mice, (**B**) left: average voluntary movements after novelty stress in the dark phase, right: the 24-h changes after novelty stress in young and aged mice, (**C**) left: average voluntary movements after water avoidance stress (WAS) in the dark phase, right: the 24-h changes after WAS in young and aged mice. *, ***; P < 0.05, 0.001 vs. fed or age-matched basal/control mice, ##, ###; P < 0.01, 0.001 between young and aged mice, n = 8, 14–16, or 7–8.

Before examining each feeding pattern, to clarify the influence of fasting and two psychological stress exposures on regular physiological activity, locomotor activity was measured. Locomotor activity in the fasted mice was lower during the dark phase (Fig. 1A). At the basal state, aged mice displayed significantly lower locomotor activity than young mice (Fig. 1B and C). Hyper-locomotor activity was observed within 12 h in the young mice after application of the novelty stress. Moreover, in aged mice, locomotor activity was markedly higher than in aged controls after exposure to the novelty stress (Fig. 1B: the effect of stress × age; F [1, 28] = 19.40, P < 0.001, and the increased ratio of young; 1.64 ± 0.09-fold, aged; 4.13 ± 0.66-fold, P < 0.001). In contrast, locomotor activity in young mice after WAS exposure was significantly lower than in the control young mice. A decrease in locomotor activity was observed in the aged-WAS mice compared to that observed in the young-WAS mice (Fig. 1C).

### Meal patterns by fasting

**Figure 2:**
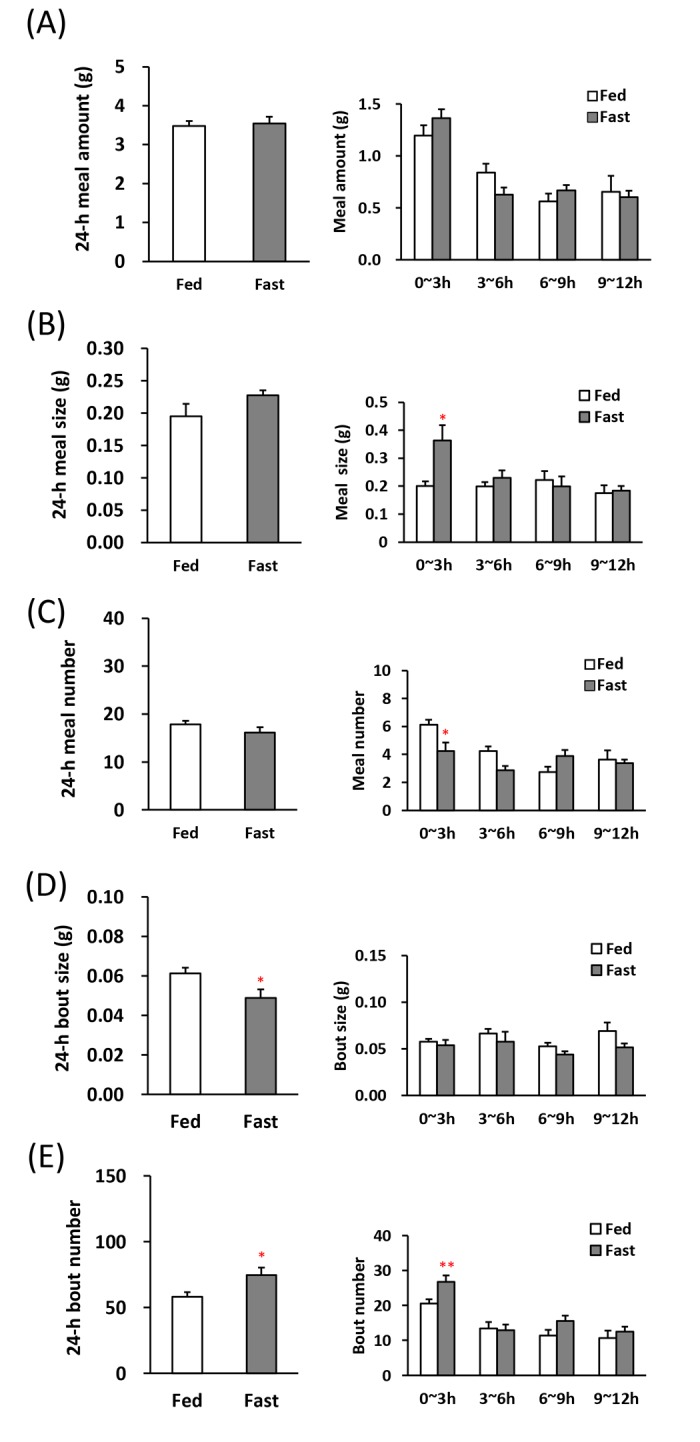
Microstructure analysis after 24-h fasting in young mice (**A**) left: the 24-h meal amount, right: meal amount per 3 h in the dark phase, (**B**) left: the 24-h meal size, right: meal size per 3 h in the dark phase, (**C**) left: the 24-h meal number, right: meal number per 3 h in the dark phase, (**D**) left: the 24-h bout size, right: bout size per 3 h in the dark phase, (**E**) left: the 24-h bout number, right: bout number per 3 h in the dark phase. *, **; P < 0.05, 0.01 vs. fed, n = 8.

First, we were analyzed the feeding pattern after fasting in order to be a control of comparison as a high-appetite state [19, 20]. The 1^st^ meal amount and time, an indicator of motivation for feeding [20] were longer and the latency to the 1^st^ meal was shorter in the fasted mice (Table 1). Although the 24-h meal amount, meal size, and meal number in between the 24-h fasted mice and the freely fed mice was no significant difference (Fig. 2A–C, left), 24-h bout size obviously decreased and bout number increased (Fig. 2D, E, left). Furthermore, in the analysis of 0–3 h, which is the initial stage of the beginning of the dark period, the meal size increased in the fasted mice and conversely the meal number decreased (Fig. 2B, C, right). The bout number was increased (Fig. 2E, right).

**Table 1:** The 1^st^ meal parameters in fed/24-h fasted mice

	Fed	Fast
1^st^ meal amount (g)	0.16 ± 0.07	0.80 ± 0.09 ^**^
1^st^ meal time (sec)	827.88 ± 348.29	3215.75 ± 523.90 *
Latency (sec)	461.13 ± 120.79	203.50 ± 54.91

*, **; P < 0.05, 0.01 vs. fed, n = 8

### Meal patterns by novelty stress

**Figure 3:**
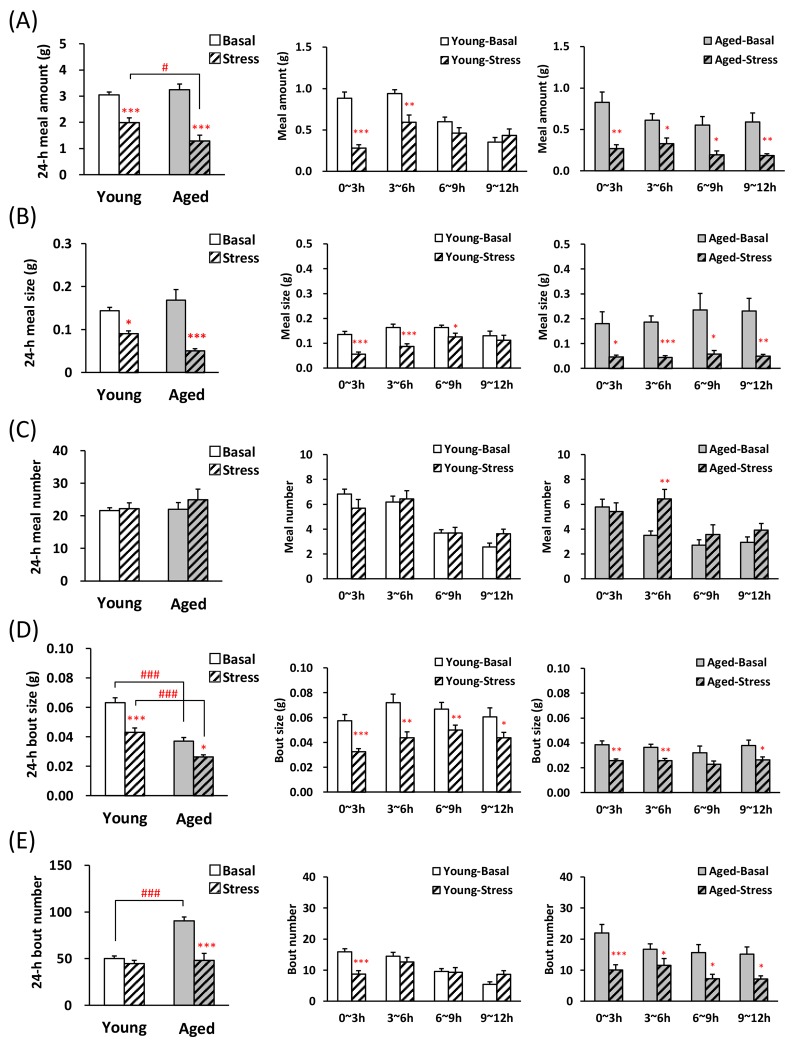
Microstructure analysis of young and aged mice after novelty stress exposure (**A**) left: the 24-h meal amount, middle and right: meal amount per 3 h in young and aged mice on dark phase, (**B**) left: the 24-h meal size, middle and right: meal size per 3 h in young and aged mice on dark phase, (**C**) left: the 24-h meal number, middle and right: meal number per 3 h in young and aged mice on dark phase, (**D**) left: the 24-h bout size, middle and right: bout size per 3 h in young and aged mice on dark phase, (**E**) left: the 24-h bout number, middle and right: bout number per 3 h in young and aged mice on dark phase. *, **, ***; P < 0.05, 0.01, 0.001 vs. age-matched basal mice, #, ###; P < 0.05, 0.001 between young and aged mice, n = 14–16.

We conducted an evaluation of the feeding pattern on the novelty stress model, which has already demonstrated that meal amount significantly reduced by stress loading and food intake is greatly affected by aging [13]. Although decrease in the 1^st^ meal amount and time was observed in both young and aged mice after stress loading (Table 2), aged mice showed a more large decrease ratio (decrease ratio of 1^st^ meal time: young; 78.4 ± 4.5 %, aged; 94.1 ±1.8 %, P < 0.01, decrease ratio of 1^st^ meal amount: young; 68.1 ± 6.8 %, aged; 71.5 ± 2.7 %). The latency to eat the 1^st^ meal significantly increased in the young and aged mice after stress exposure (Table 2). Fig. 3A and B reveal that the 24-h meal amount and size were significantly lower in young and aged mice after exposure to novelty stress than in those acclimated to the device (basal mice), although basal values in both young and aged mice was similar levels. Observation of changes in meal amount and meal size in the dark period revealed that in young mice, these decreases after stress did not persist for up to 12 h, but in aged mice it lasted up to 12 h (Fig. 3A, B, middle and right). Although, no differences in 24-h meal number were observed in basal and stressed mice, the meal number of aged mice during dark phase increased only in 3–6 h (Fig. 3C, right). Twenty four hour bout size in aged-basal or stressed mice was significantly lower compared with young (Fig. 3D, left) and bout size in the dark period decreased or tended to decrease up to 12 h in both young and aged mice after the stress (Fig. 3D, middle and right). In the aged-basal mice, 24-h bout number was significantly higher than in young-basal mice and the application of novelty stress decreased the 24-h bout number only in aged mice (Fig. 3E, left). The bout number in young mice 0–3 h after the stress significantly decreased, compared with basal young mice. It returned to the basal level at 3 h, and increased conversely during 9–12 h (Fig. 3E, middle). In aged mice, the bout number decreased significantly during the dark period after stress exposure (Fig. 3E, right).

**Table 2:** The 1^st^ meal parameters in basal/novelty stressed young and aged mice

	Young basal	Young stress	Aged basal	Aged stress
1^st^ meal amount (g)	0.14 ± 0.03	0.05 ± 0.01 ^**^	0.11 ± 0.04	0.03 ± 0.00 *
1^st^ meal time (sec)	724.88 ± 175.43	156.31 ± 32.39 ^**^	1027.93 ± 390.08	61.07 ± 18.68 *
Latency (sec)	864.56 ± 155.32	3069.56 ± 528.26 ^**^	1847.71 ± 517.09	4189.71 ± 548.44 ^**^

*, **; P < 0.05, 0.01 vs. age-matched basal mice, n = 14–16

### Meal patterns by WAS

**Figure 4:**
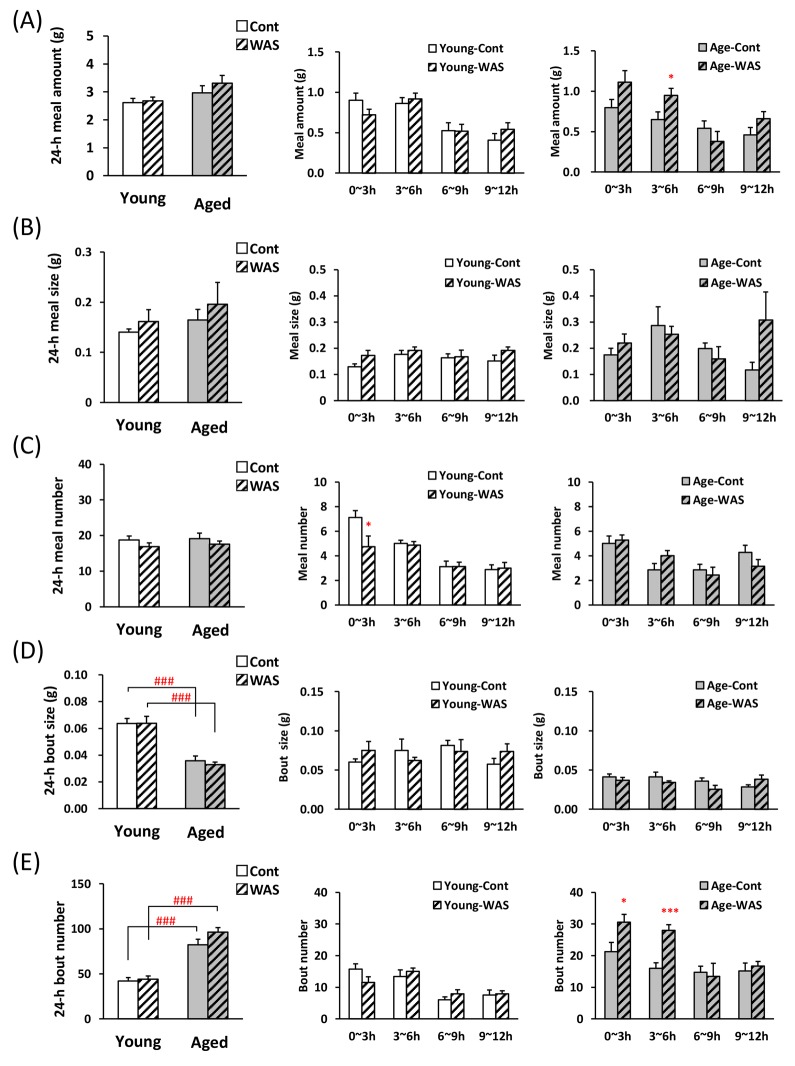
Microstructure analysis of young and aged mice after water avoidance stress exposure (**A**) left: the 24-h meal amount, middle and right: meal amount per 3 h in young and aged mice on dark phase, (**B**) left: the 24-h meal size, middle and right: meal size per 3 h in young and aged mice on dark phase, (**C**) left: the 24-h meal number, middle and right: meal number per 3 h in young and aged mice on dark phase, (**D**) left: the 24-h bout size, middle and right: bout size per 3 h in young and aged mice on dark phase, (**E**) left: the 24-h bout number, middle and right: bout number per 3 h in young and aged mice on dark phase. *, ***; P < 0.05, 0.001 vs. age-matched control mice, ###; P < 0.001 between young and aged mice, n = 7–8.

It was examined whether the stress load affects bout parameters in the WAS model which is recognized not to affect total food intake in the previous report [21, 22]. No significant changes were observed in the 1^st^ meal amount, time, and latency by WAS in young and aged mice (Table 3). WAS exposure did not make changes the 24-h meal amount (Fig. 4A, left), meal size (Fig. 4B), and meal number (Fig. 4C) in young and aged mice. The 24-h bout size in the aged control mice was significantly lower than in the young control mice (Fig. 4D), and the bout number in the aged control mice was significantly higher than in the young control mice (Fig. 4E).

**Table 3:** The 1^st^ meal parameters in control/WAS young and aged mice

	Young control	Young WAS	Aged control	Aged WAS
1^st^ meal amount (g)	0.11 ± 0.03	0.09 ± 0.02	0.12 ± 0.02	0.22 ± 0.04
1^st^ meal time (sec)	503.13 ± 123.37	411.50 ± 105.29	1296.71 ± 445.13	901.14 ± 167.31
Latency (sec)	652.00 ± 278.09	735.00 ± 437.42	542.86 ± 86.32	725.00 ± 256.44

n = 7–8

The meal pattern during dark phase was analyzed by 3-h time points after WAS. Each 3 h meal amount in young mice was unchanged due to WAS stress compared with control mice (Fig. 4A, middle). In contrast, a tendency to increase and an increase in the 0–3 h and 3–6 h meal amount following WAS in aged mice was observed (Fig. 4A, right). The meal size was unchanged in young and aged mice exposed to WAS compared with control (Fig. 4B, middle and right). Only in young mice at 0–3 h, the meal number decreased (Fig. 4C). The 0–6 h bout number in aged mice exposed to WAS were significantly higher than in the control mice (Fig. 4E, right), although no change in the bout size was observed in young and aged mice after WAS exposure (Fig. 4D middle and right).

## DISCUSSION

The present study demonstrated that two kinds of psychological stress induced different alterations in meal pattern. The exposure of novelty stress decreased meal amount in both young and aged mice, and also decreased meal size, bout size, and bout number. In addition, these parameters in the dark period were persistently observed in aged mice. On the other hand, the WAS load clearly had a characteristic effect on the bout number of aged mice, and it showed an increase in the meal amount and a further increase in the number of bouts.

Locomotor activity of fasted mice decreased significantly simultaneously with the onset of the dark period. Also, it had a negative correlation with meal amount and meal time (Supplementary Fig. S1). In addition, the meal number declined at the same timing as locomotor activity decrease in fasted mice. As one hypothesis, it was thought that the cause of this decrease in activity was due to staying at the feeding station and eating food. An increase in locomotor activity has been observed in isolation stress-loaded and chronic unpredictable mild stress-loaded rodents [23, 24]. Thus it is likely that it means that rodents elevated locomotor activities due to anxiety or exploratory behavior. In addition, locomotor activity may be susceptible to aging. The locomotor activity of the aged control mice was clearly lower than that of the young mice. It was higher than the young mice during light phase, although the dark phase are generally the active period of rodents, thereby indicating a disruption in the circadian rhythm of the aged mice (Supplementary Fig. S2).

Interestingly, the low basal locomotor activity of aged mice was markedly increased by novelty stress more than it was increased in the young mice, consequentially resulting in the same locomotor activity level as that observed in the young mice. Since the behavior of aged mice increased to the same extent as young mice after stress, it seems that the decrease in locomotor activity in control aged mice was not due to gait functional impairment.

**Figure 5:**
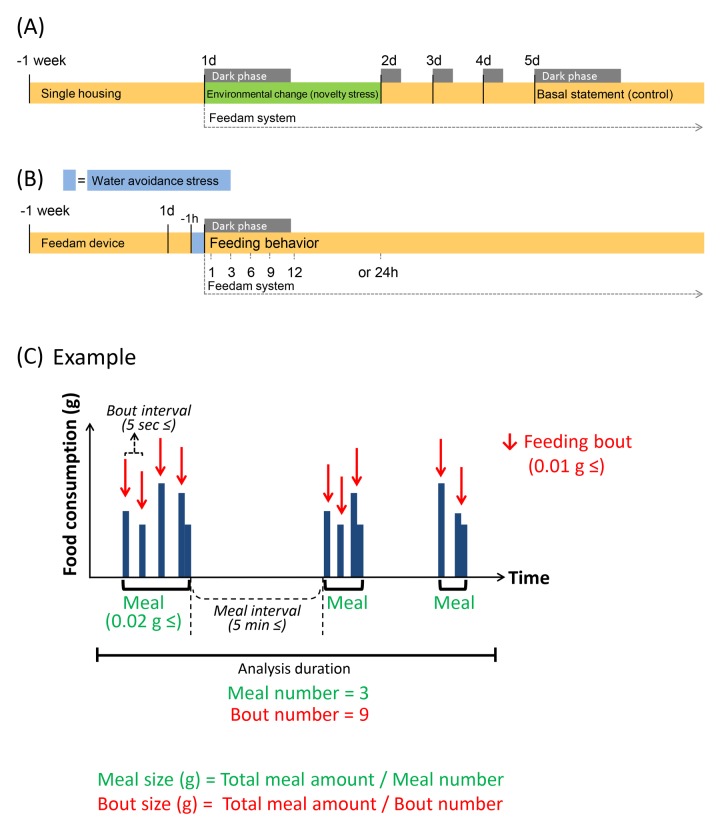
Experimental schedule and schematic feeding bout (**A** and **B**) It is shown experimental schedule. (**C**) Green indicates “Meal” parameters, and red indicates “Bout” parameters. It is shown a chart when meal number is 3 and bout number is 9 as an example. Each value indicates the number of times per analysis time. Feeding bout was defined as a new bout when the time without feeding was longer than 5 sec from the previous response and was equal to or greater than 0.01 g. Food consumption was considered to be a “Meal” when the feeding bouts occurred within 5 min of the previous response and the sum of the food consumed during the bout was equal to or greater than 0.02 g; when the feeding bouts were longer than 5 min apart, these were considered to be a new meal.

Mammals, including rodents, ingest a desired amount of food by repeating small feeding behaviors such as nibbling and swallowing within a short amount of time (Fig. 5C). Therefore, to determine the biological sig-nificance of the bout parameters by evaluating the influence of aging on the minimum feeding behaviors in stress-loaded mice, we defined a minimum feeding be-havior as a bout and focused on meal patterns, including not only the meal amount but also the bout parameters. The meal pattern in a high-appetite state induced by 24-h fasting is shown in Fig. 2. These changes may be considered to be a typical feeding pattern during an orexigenic state. The 1^st^ meal amount and time were markedly prolonged, and the latency to eat the 1^st^ meal was shortened by fasting. These changes seem to be related to the increased feeding motivation based on the knowledge so far [20]. When was analyzed at the beginning of dark phase (0–3 h), meal size and bout number was increased under fasting, although meal amount change was not observed. The orexigenic feeding pattern due to fasting may be characterized primarily by not only increasing motivation parameters, but also increasing meal size and bout number. Meal size and meal number are closely related to satiation and satiety, respectively [25]. An increase in meal size is defined as a decrease in satiation, meaning that feeding termination dose not to occur for a longer period of time. This was thought to reflect the orexi-genic state. In contrast, a decrease in meal number may be defined as an increase in satiety, meaning a later initiation of a new meal after one meal is completed. The conflicting result of increasing the meal size and decreasing the meal number in fasted mice is likely to mean a situation that mice may show an uninterrupted feeding behavior, meaning a state in which is focused on eating. Interestingly, the meal number was decreased and the bout number was increased by fasting. This suggests that increased appetite may be mediated by increasing meal size via an increase in bout number, which is the smallest feeding behavior, nibbling and swallowing, rather than the number of meals. In addition, the 24-h bout size significantly decreased, but no change was observed immediately after the beginning of dark phase with high appetite, so it was inferred that the decrease in bout size may not directly correlate with appetite.

The novelty stress exposed to young mice showed a decrease in meal size at 0–9 h after the start of stress, although the meal number did not change. This change clearly shows an increased satiation (earlier termination of a meal) and may mean a typical anorexic state [25]. The meal pattern of aged mice after exposure to novelty stress was characterized by sustained lower meal amount and size than that of young. Novelty stress induces a prominent and sustained decrease in food consumption in aged mice [13], which coincides with the results of the present study that were obtained using an automated monitoring device. We have already proved that aged mice maintain food intake by increasing bout number [18]. This microstructure study indicated that unlike young mice, the characteristic of sustained feeding suppression in stress-loaded aged mice may be thought to be due to a decrease in the number of minimum feeding behavior such as licking, nodding, swallowing. Additionally, it is likely that aged mice fail to challenge larger bout size duo to additional gastrointestinal damages when novelty stress was exposed to aged mice. In the stress-loaded aged mice, the 3–6 h meal number remained increased despite the reduced food intake compared with the control mice. This cause is unknown, and further detailed examina-tion is necessary.

Previous reports have demonstrated that the exposure of WAS to mice results in no effect on food intake [21, 22], which was in agreement with the results of the present study based on manual measurements (Supplementary Fig. S3) and 24-h meal parameters measured by the automated monitoring device (Fig. 4). We expected the possibility of detecting the influence of WAS exposure on meal pattern without change of meal amount. Interestingly, unlike novelty stress, the meal pattern after WAS was clearly different between aged and young mice. The meal number in young mice after the stress exposure was significantly decreased compared with control mice, although the meal amount was not altered. It seems likely that exposure of WAS to young mice increased satiety. The meal amount in aged mice after the WAS was higher than that of control, although the feeding motivation was unchanged. These data suggest that the increase in meal amount of WAS-loaded aged mice may not be caused by increased appetite. In addition, the exposure of WAS caused a further increase in the bout number compared to the control aged mice without altering other parameters. It may be directly involved in an increase in meal amount in WAS-loaded aged mice. It seems likely that an increase in meal amount via an increase in the number of bouts may reflect overeating by stress in aged mice. Thus, the evaluation of bout number was considered to be a good tool to capture the characteristic feeding patterns of aged mice, which had never been observed before.

Recently, the relationship between changing feeding pattern and feeding related peptide such as ghrelin, nesfatin-1, and peptide YY (PYY) has been reported [9, 18, 26]. We already demonstrated that increase in peripheral PYY concentrations are partly involved in characteristic feeding patterns such as an increase in the number of bouts and a decrease in size in aged mice [18]. On the other hand, there is no evidence on fasted feeding patterns in rodents, especially bout parameters. In fasted mice, the peripheral ghrelin increases, conversely the anorexic factor such as leptin and cholecystokinin (CCK) is considered to be suppressing. In aged mice, besides high plasma PYY levels [18], abnormality of many feeding-related factors such as hyperghrelinemia, hyperleptinemia, hyperinsulinemia is exhibited under freely fed condition [27]. In addition, stress-loaded aged mice may further modify such as hormone abnormalities [5, 14]. Intracerebroventricular administration of ghrelin to normal young mice does not affect meal size, although it causes an increase in meal number (frequency) [28]. In the growth hormone secretagogue receptor (GHSR)-KO middle aged mice, a decrease in meal number has been observed [9], but data on bout has not been reported. The meal number in fasted mice was rather similar to the meal pattern in the GHSR-KO mice [9]. The difference in these experimen-tal results may depend on the influence of functional changing the age-related factors by GHSR-KO and the age of animal used. The exposure of novelty stress to mice causes a decrease in peripheral ghrelin, which causes a reduction in feeding [5, 14], however: the results of meal pattern in the current study clearly differed from that of aged GHSR-KO mice [9]. Our results were similar to the typical feeding patterns of anorexic factor administration by CCK [12] and nesfatin-1 [26]. Treatment with CCK and nesfatin-1 was consistent with our novelty stress in that it caused a significant decrease in bout number. From these findings, when exploring detailed mechanism of appetite abnormality, evaluation of not only food intake but also meal pattern and bout parameter may be useful for elucidation of more accurate mechanism.

In WAS stress, an increase in peripheral ghrelin [29] and an increase in central nesfatin-1 [30, 31] have been confirmed. Intracerebroventricular administration of nesfatin-1 causes decreases in meal and bout number without affecting meal size [26]. It was similar results that WAS-loaded young mice caused decreasing the meal number without changing meal size. On the other hand, increased meal amount and further increase in bout number in aged mice was observed. In this study, we did not measure central nesfatin gene or blood hormone concentration in WAS mice, but aging and stress can greatly influence the secretion of these feeding related factors. Also as these feeding-related peptides and neurotransmitters cross-talk to each other, changes in the meal pattern due to single peptide administration may only reflect some of our results. In addition, in studies with guinea pigs and rats, since WAS was induced stomach contraction [32] and inhibition of adaptive relaxation via 5-HT_2B_R activation in the stomach [33], it seems likely that this gastric dysfunction was also related to an increase in the number of bouts in our study. In order to clarify the relationship between feeding-related factor and meal pattern, we further considered that it is necessary to analyze a wide range of eating-related peptides in the brain and periphery and simultaneous analysis of feeding pattern.

In conclusion, these findings revealed that changes in bout number may lead to abnormal stress-related feeding patterns in aged mice. In addition to the meal parameter, the evaluation of the bout number will help to elucidate the mechanism of eating abnormalities in aging.

## METHODS

### Animals

Male C57BL/6J mice (9 weeks and over 24 months old, Charles River, Yokohama, Japan) were used in this study. Before the experiments, the mice were placed in a single housing unit under controlled temperature, humidity, and illumination (5:00 h–17:00 h) and then transferred into food intake monitoring cages as detailed below for at least 5 days. Mice were provided a standard rodent diet (Oriental Yeast Co., Ltd., Tokyo, Japan) and water *ad libitum*. All animals were humanely euthanized by exsanguination under isoflurane anesthesia at the end of the experiment. The Tsumura Animal Care and Use Committee (permit no. 15-040) approved all protocols used in this study.

### Induction of stress

The load of novelty stress was slightly modified as previously described [13, 14]. Here, we defined mice as under “environmental change stress” upon transfer from the home cage to the food intake monitoring device (on Day 1) and as under a “basal state” on Day 5 after the transfer to the device (Fig 5A). The protocol was decided based on changes in stress hormones after environmental stress [5], as previously reported [12] (mice usually habituated to the new environment within 3–4 days and showed normal food intake and regular body weight gain) and the results from monitoring daily food intake and body weight.

The procedure for WAS was essentially conducted as previously described [34]. Exposure to WAS was performed by placing a mouse on a platform (cylinder with a diameter of 5 cm) in the center of a plastic container (inner diameter D × W × H: 27 cm × 35 cm × 21.5 cm, IRIS OHYAMA Inc., Sendai, Japan) filled with water for 1 h. The mice were exposed to the stress for 1 h immediately before feeding measurements (16:00 h–17:00 h). After the stress procedure, the mice were immediately returned to the device, and we monitored the feeding behavior simultaneously with the onset of the dark phase (17:00 h, Fig. 5B). The mouse body weight (BW) was monitored daily before each stress exposure, and the percentage of the daily individual weight change was calculated as follows: ([BW_Day x −_ BW_Day 0_] / BW_Day 0_) × 100 (Supplementary Fig. S3).

### Automated food intake monitoring

The microstructure analysis of feeding behavior was conducted using a High Performance Food Intake Monitor device (Feedam system, cFDM-300ASH, Melquest Ltd. Toyama, Japan) for mice, which allows for continuous monitoring of meal patterns in undisturbed mice with minimal human interference [12, 26]. Mice were acclimatized to the device environment for at least 5 days and displayed normal food intake and regular BW gain. The experimental protocol of this study is shown in Fig. 5.

The automated system weighs the food per second (± 0.01 g) and algorithmically detects “not eating” as stable weight and “eating” as unstable weight. As previous reports [12, 18], a feeding bout (as indicated in Fig. 5C) was defined as a new bout when the time without feeding was longer than 5 sec from the previous response and was equal to or greater than 0.01 g. A feeding bout is recorded as the minimum feeding behavior with a start time, duration, and the amount of food consumed. Food intake was considered to be a meal when the feeding bouts occurred within 5 min of the previous response and the sum of the food consumed during the bout was equal to or greater than 0.02 g; when the feeding bouts were longer than 5 min apart, these were considered to be a new meal [12]. The meal parameters included the total meal amount, time, meal size, meal duration, and number. The bout parameters included the bout size, bout duration, and number (Supplementary Table S1). For the purpose of clarifying the correctness of the setting conditions of our system, we compared the result of meal amount by automatic measurement with the manual by using the novelty stress model which the negative influence of feeding was demonstrated in the previous report, and confirmed that the result of each was consistent (Supplementary Table S2). As an index of appetite motivation, the first time and amount of food ingested from the beginning of the feeding pattern measurement were also recorded as the 1^st^ meal amount, 1^st^ meal time, and latency to eat the 1^st^ meal [19, 20]. Locomotor activity was monitored using a thermal-radiation motion detector (AS-10, Melquest Ltd.), the activity sensor module of the Feedam apparatus attached to the top of the apparatus [35]. Locomotor activity was continuous-ly recorded every 1 min on a computer. The data were calculated by accumulating the count per 1 h and were quantified by dividing them into dark and light periods, which correspond to the active and resting periods for mice, respectively. These parameters were calculated using the Feedam-BM software (Melquest Ltd.). Supplementary Fig. S4 shows the typical pattern of the meal amount and the voluntary movement at each stress exposure.

### Statistical analysis

Data were expressed as the mean ± SEM. Pairwise differences between groups were analyzed using Student's t-test or Paired t-test. Differences between young and aged groups were analyzed using two-way analysis of variance (ANOVA) followed by the Tukey–Kramer *post hoc* test. Differences were considered statistically significant when P < 0.05.

## SUPPLEMENTARY MATERIALS FIGURES


