# Supplementary material for: Psychological stress exposure to aged mice causes abnormal feeding patterns with changes in the bout number
Source: Aging (Albany NY). 2017 Nov 9;9(11):2269–87. doi: 10.18632/aging.101320 (PMC5723686; doi:10.18632/aging.101320)
Supplement: Supplementary file 1 [file aging-09-2269-s001.pdf]

SUPPLEMENTAL MATERIAL

Supplementary Table S1. Parameters of feeding pattern in microstructure study.

Parameters	Definition
Meal amount (g)	Food intake for analysis duration
Meal number	Number of meal for analysis duration
Meal size (g)	Meal amount / Meal number
Bout number	Number of bout for analysis duration
Bout size (g)	Meal amount / Bout number
1st meal amount (g)	Food intake at the 1st Meal
1st meal time (s)	Time taken for the 1st Meal
Latency (s)	Time from measurement start to the 1st Meal start
Meal time (s)	Total time taken for meal
Meal duration (s)	Meal time / Meal number

Supplementary Table S2. Comparison of manual measurement of meal amount based on previously reported method with automatic measurement of meal amount by using Feedam-system in two types of psychological stressed mice.

Methods	Basal	Novelty stress	P value	Decrease rate
Manual measurement	3.02 ± 0.06 g	2.18 ± 0.20 g	P < 0.001	27.8 ± 6.5 %
Feedam measurement	3.05 ± 0.10 g	1.99 ± 0.18 g	P < 0.001	34.8 ± 6.0 %

Methods	Young-Control	Young-WAS	Aged-Control	Aged-WAS
Manual measurement	2.83 ± 0.17 g	2.84 ± 0.11 g	3.35 ± 0.23 g	4.03 ± 0.30 g
Feedam measurement	2.62 ± 0.15 g	2.68 ± 0.14 g	2.97 ± 0.26 g	3.31 ± 0.28 g

Meal amount by manual measurement and meal amount by Feedam-system measurement for 24 h were compared. Basal/Novelty stress was performed with young mice (n = 16). Control/water avoidance stress (WAS) was performed with young (n = 8) and aged mice (n = 7). Data are presented as mean ± SEM.

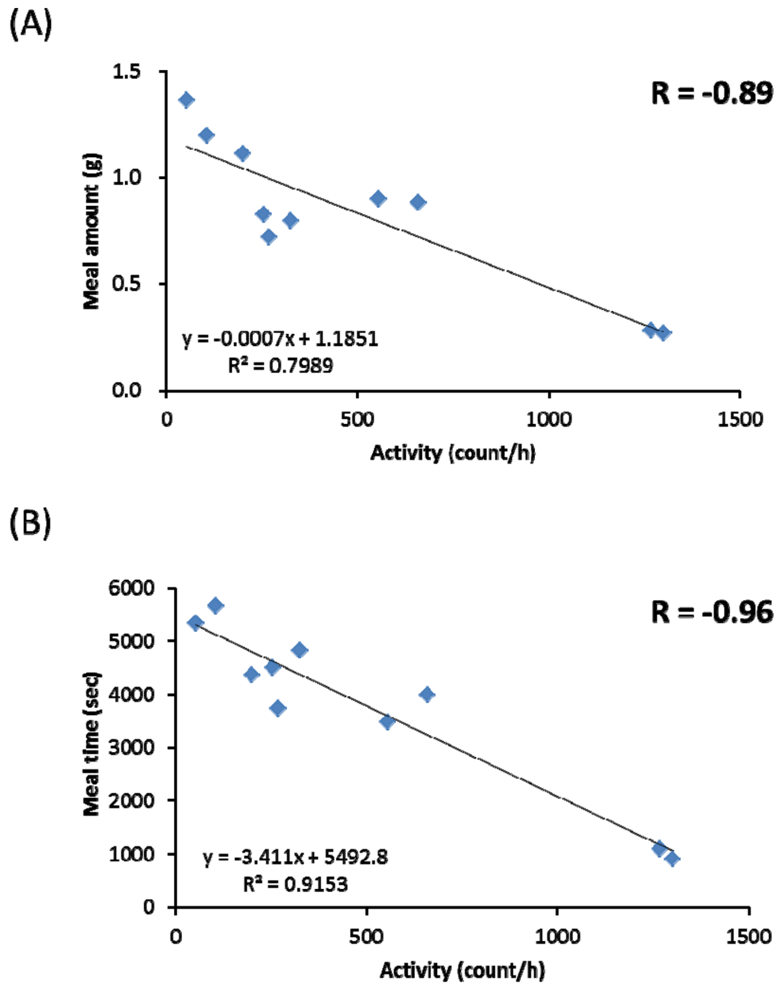

Supplementary Figure S1. Correlation between locomotor activity and meal amount/meal time. We analyzed the correlation between locomotor activity and meal amount/meal time in 0–3 h on dark phase. The average value of each group (fed, fast, young-basal, young-novel, aged-basal, aged-novel, young-control, young-WAS, aged-control, aged-WAS) is shown. (**A**) Correlation diagram between locomotor activity and meal amount. $P < 0.001$ by Peason correlation test. (**B**) Correlation diagram between locomotor activity and meal time. $P < 0.001$ by Peason corellation test.

(A)

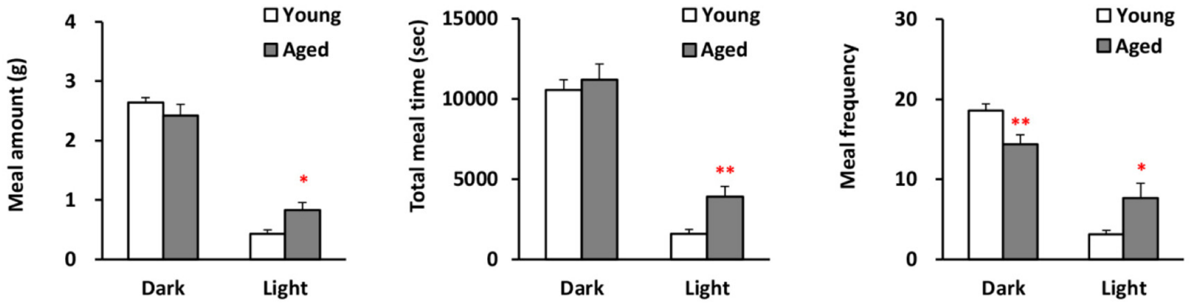

(B)

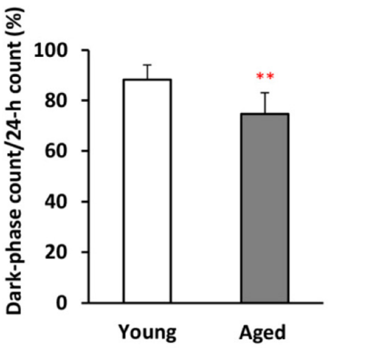

Supplementary Figure S2. Meal parameters in the dark/light phase and ratio of locomotor activity in the dark phase in young and aged mice. (**A**) Meal amount, Total meal time, and Meal frequency. (**B**) Ratio of locomotor activity in the dark phase. *, **; P < 0.05, 0.01 vs. young mice, n = 14–16.

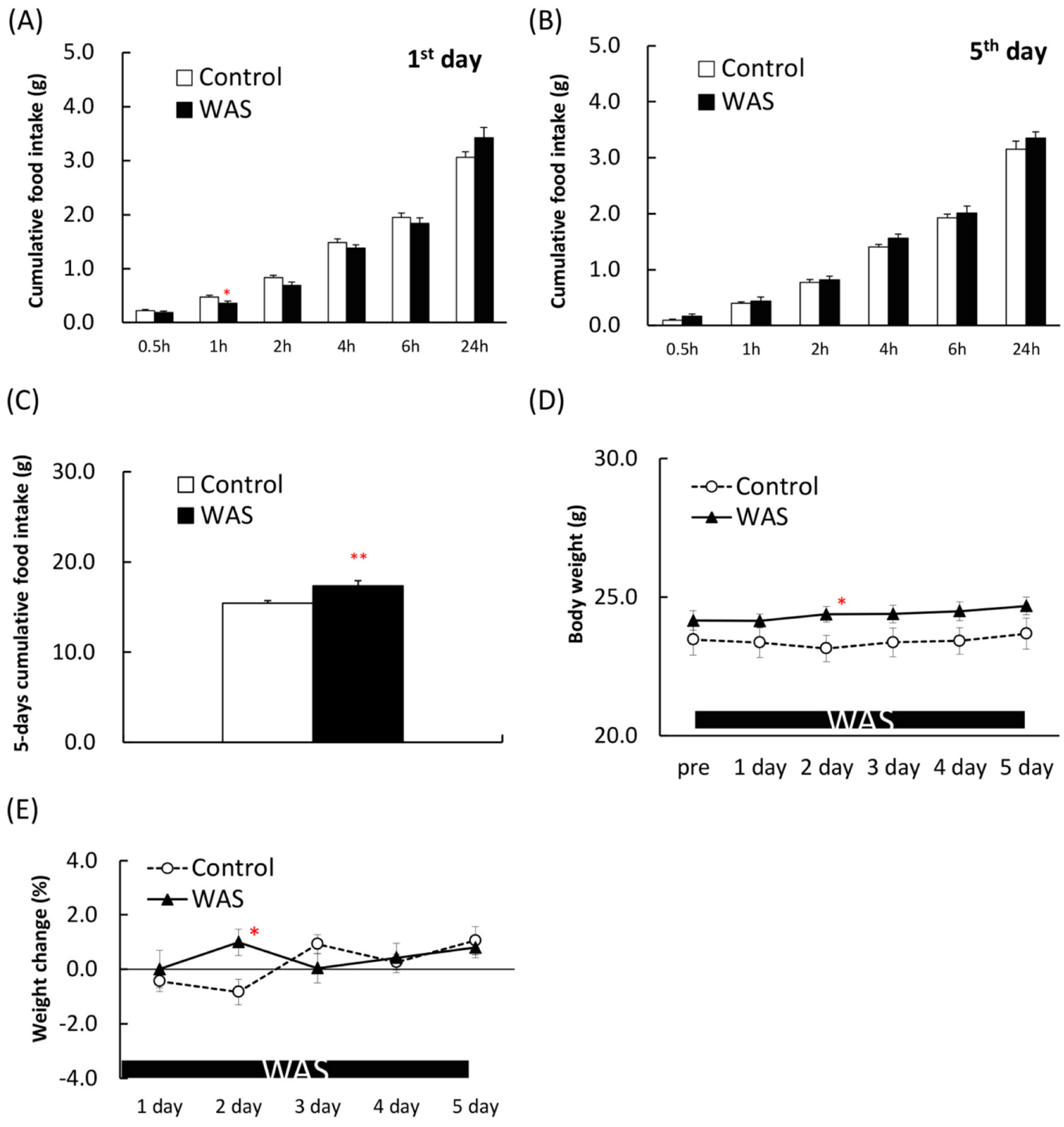

Supplementary Figure S3. Changes in food intake and body weight in young mice after water avoidance stress exposure (for consecutive 5-days) by manual measurement. (**A**) Cumulative food intake on the WAS-1st day, (**B**) Cumulative food intake on the WAS-5th day, (**C**) 5-days cumulative food intake, (**D**) Body weight changes, (**E**) Body weight gain (%). *, **; P < 0.05, 0.01 vs. control, n = 10. Water avoidance stress: WAS.

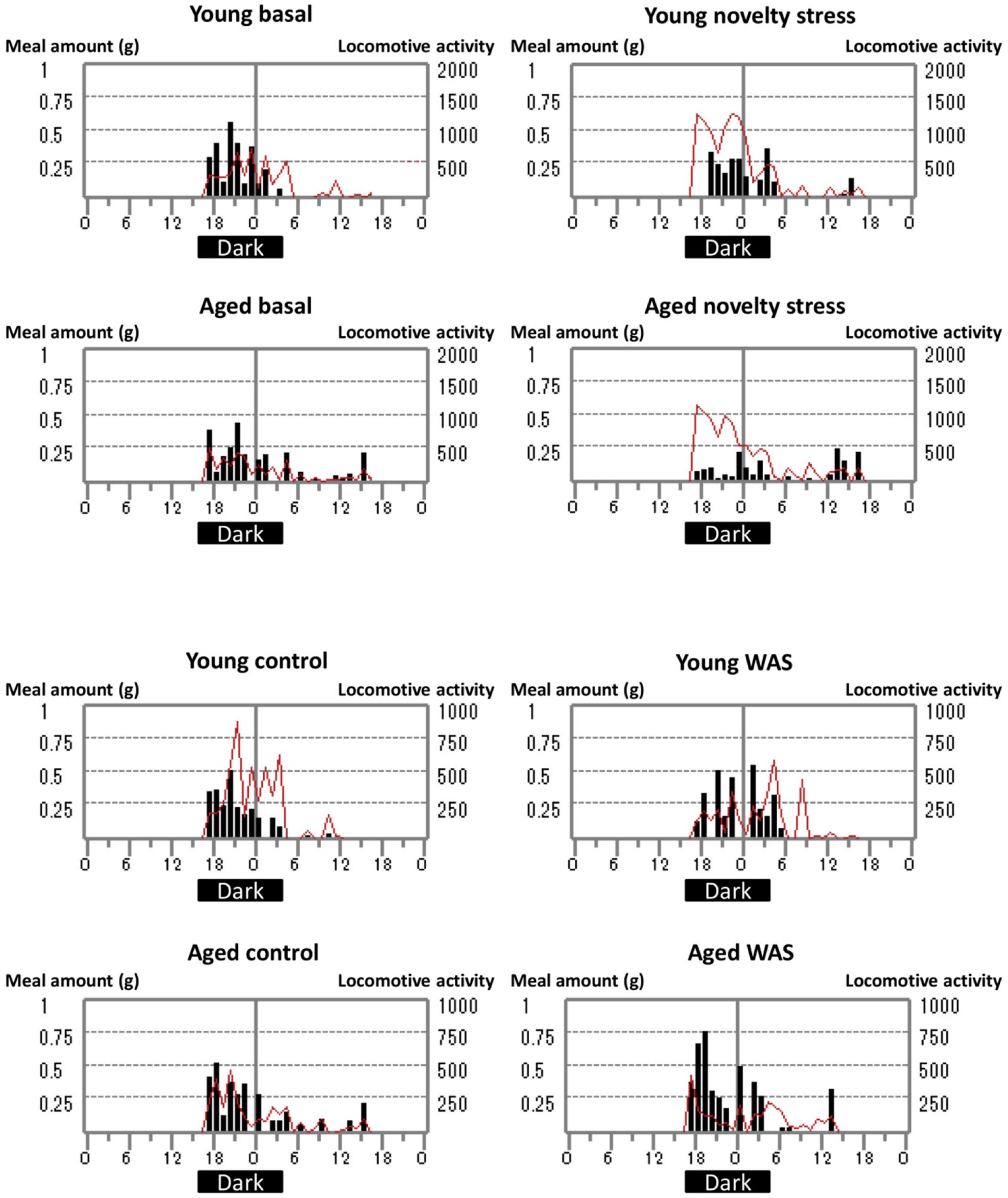

Supplementary Figure S4. Typical meal amount and voluntary movement changes detected by the Feedam system.

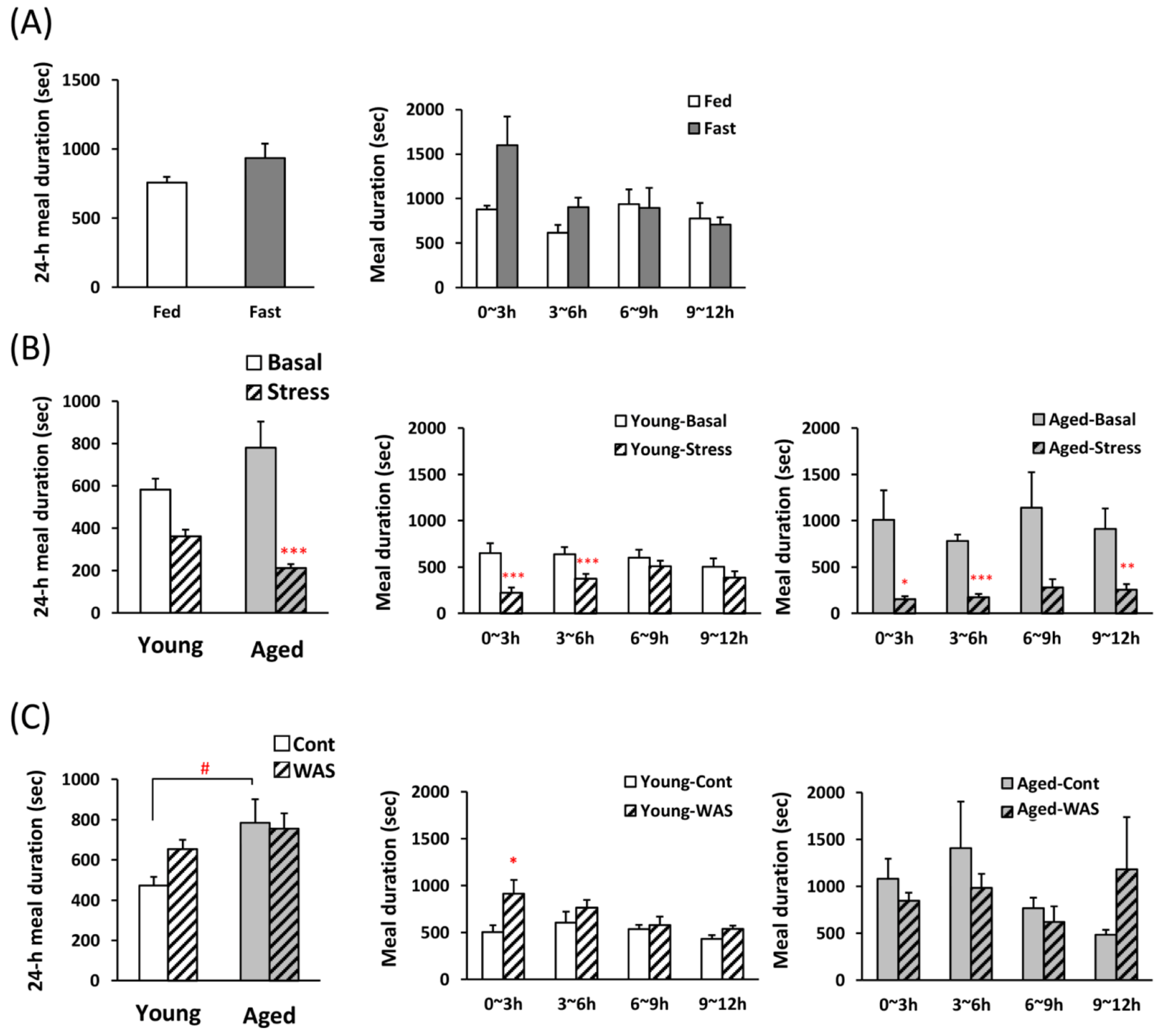

Supplementary Figure S5. Changes in meal duration in young and aged mice after fasting or exposure to novelty stress/water avoidance stress. (**A**) left: the 24-h meal duration, right: meal duration per 3 h on dark phase after 24-h fasting in young mice, (**B**) left: the 24-h meal duration, middle and right: meal duration per 3 h on dark phase in young and aged mice after novelty stress, (**C**) left: the 24-h meal duration, middle and right: meal duration per 3 h on dark phase in young and aged mice after water avoidance stress (WAS). *, **, ***; $P < 0.05$, 0.01, 0.001 vs. age-matched basal/control mice, #; $P < 0.05$ between young and aged mice, n = 8, 14–16, or 7–8.
